# Emigration preferences and plans among medical students in Poland

**DOI:** 10.1186/1478-4491-10-8

**Published:** 2012-04-30

**Authors:** Krzysztof Krajewski-Siuda, Adam Szromek, Piotr Romaniuk, Christian A Gericke, Andrzej Szpak, Krzysztof Kaczmarek

**Affiliations:** 1 Department of Health Policy, School of Public Health, Medical University of Silesia, Katowice, Poland; 2 Department of Health Promotion, Institute of Public Health, Faculty of Health Sciences, Collegium Medicum, Jagiellonian University, Cracow, Poland; 3 Department of Computer Science and Econometrics, Faculty of Organisation and Management, Silesian University of Technology, Gliwice, Poland; 4 Peninsula CLAHRC, National Institute for Health Research, Peninsula Medical School, Universities of Exeter and Plymouth, Plymouth, United Kingdom; 5 Department of Public Health, Faculty of Health Sciences, Medical University of Bialystok, Bialystok, Poland; 6 Department of Health Policy, Faculty of Public Health, Medical University of Silesia, ul. Piekarska 18, 41-902, Bytom, Poland

**Keywords:** Medical doctors, Medical students, Emigration, Professional mobility, Poland

## Abstract

**Background:**

Migration and ethical recruitment of health care workers is receiving increased attention worldwide. Europe’s aging population is creating new opportunities for medical doctors for finding employment in other countries, particularly those of a better standard of living.

**Methods:**

We conducted a survey among 1214 medical students in five out of eleven universities in Poland with medical schools in October 2008. A series of statistical tests was applied to analyse the characteristics of potential migrants. Projections were obtained using statistical analyses: descriptive, multifactorial logistic regression and other statistical methods .

**Results:**

We can forecast that 26–36% of Polish medical students will emigrate over the next few years; 62% of respondents estimated the likelihood of emigration at 50%. Students in their penultimate year of study declared a stronger desire to migrate than those in the final year. At the same time, many students were optimistic about career opportunities in Poland. Also noted among students were: the decline in interest in leaving among final year students, their moderate elaboration of departure plans, and their generally optimistic views about the opportunities for professional development in Poland.

**Conclusions:**

The majority of Polish students see the emigration as a serious alternative to the continuation of their professional training. This trend can pose a serious threat to the Polish health care system, however the observed decline of the interest in leaving among final year students, the moderate involvement in concrete departure plans and the optimistic views about the opportunities for professional development in Poland suggest that the actual scale of brain drain of young Polish doctors due to emigration will be more limited than previously feared.

## Background

Migration and ethical recruitment of healthcare workers is receiving increased attention worldwide [1-5]. Europe’s aging population is creating new opportunities for Polish medical doctors, while inadequate working conditions and relatively low salaries push many high-skilled healthcare workers to search for employment abroad. The perception is that a large number of healthcare workers have migrated since Poland’s accession to the European Union (EU) in 2004, in particular to the United Kingdom and neighbouring Germany. However, there is little reliable data and limited research on the migration of Polish healthcare workers [6-8].

According to data provided by the Polish Chamber of Physicians and Dentists, between 4 May 2004 and 30 April 2005, 2533 certificates of good standing were issued, which are required in order to obtain registration as a doctor in another EU Member State. Therefore, this number can serve as an estimate for the number of doctors who left Poland for work abroad in the 12 months following its accession to the EU in 2004 [9]. Another more indirect indicator is the total number of registered doctors in Poland which has steadily decreased since EU accession: from 92 982 doctors in 2003 to 82 397 doctors in 2008 (WHO Europe Health for All Database July 2010).

### Aim of the study

As there is considerable uncertainty whether the scale of emigration will continue at the current rate, increase or decrease in coming years we conducted a cross-sectional survey to assess the most likely scale of emigration of Polish doctors and to identify the characteristics of potential migrants.

## Methods

We conducted a survey among 1214 medical students in five out of eleven universities in Poland with medical schools (Katowice, Poznań, Kraków, Warszawa, and Białystok) which all provide comparable curricula. The selection of medical schools was based on geography and socio-economic profiles to cover all major regions in Poland (i.e. rural vs. urbanised, agricultural vs. industrialised, west vs. east). The selected schools are therefore representative for the whole country. The study was conducted between October and November 2008 and included 25 questions regarding students’ emigration plans, push and pull factors, length of planned migration and target country.

Descriptive statistical analysis was conducted to calculate means, standard deviations, quartiles, variation area features as well as variation, heave, and asymmetry coefficients. The correlation analysis was performed using Pearson correlation coefficient and Spearman’s rank coefficient, while the variables of the two variants, or multi-variants, were calculated using Spearman’s rank coefficient and Yule’s factor, Chi-square, correlation relations (for relationship nominal and ordinal measures), and by the Kendall’s coefficient tau-b and tau-c [10,11].

In case of normal distribution variables, the Student-*t* test was used to determine the homogeneity of variance (Fisher-Snedecor’s and Levene’s tests) and to compare averages for independent groups. The Mann–Whitney *U* test was used to compare groups with non- normal distributions. In cases of heterogeneous variance of compared groups with a normal distribution, Cochran-Cox’s C test was used [10,12].

Distributions of the studied variables were examined using Shapiro-Wilk’s, Chi-square and Kolmogorov-Smirnov’s tests [13,14]. Statistical significance was assumed at α ≤ 0.05.

Forecasts were estimated by applying statistical analyses, such as descriptive analysis, but also multivariate logistic regression and log-linear analysis [15-20].

## Results

### Characteristics of the sample

The study included 1214 students. Respondents were asked to fill in the questionnaires shortly after lectures (before leaving the lecture theaters), which resulted in a response rate of 100% (in relation to the selected group of respondents) and high representativeness of the sample (in relation to the total number of students). However, 37 incomplete questionnaires were excluded from the analysis resulting in 1177 questionnaires examined in this paper. The final number represents 46.5% of all 5^th^ and 6^th^ year students at the participating medical schools. 47.5% of the study participants were 5^th^ year students, while 52.5% were 6^th^ year students. Females constituted two-thirds of the sample. Most respondents hail from urban environments; 42.3% are from large cities and 45.5% are from medium-size and small cities; 12.2% come from rural areas. The majority of the students’ parents – 56.7% of fathers and 60.8% of mothers — are university-educated. Respondents ranged in age from 20 to 35 years, with 95% being between 22 and 26 years of age. The vast majority (77.2%) desire to work in hospital settings. Additional 13.8% aspire to open a private practice, while 4.3% and 3.7% want to work in out-patient or family medicine, respectively.

### Desire to emigrate

62.1% of respondents plan to seek employment abroad after graduation. Respondents were asked to estimate the probability of migrating on a scale from 0 to 100. The average probability of emigration was 50% (± 26.8%) with no significant difference between males and females (*p* < 0.0001). Every fourth student contemplating migration specified the probability of leaving at 30% or less (Q1 – lower quartile). Three out of every four respondents assessed the likelihood of departure at 70% or less (Q3 – upper quartile). One third planned to leave one year after graduation, 17.2% planned to leave within 3 to 5 years after graduation and 14.2% in more than five years after graduation. Of those, 44% who declared a probability of emigration of at least 5% contemplated long-term or permanent emigration. 35% planned to stay abroad a few years and 15.5% planned to stay abroad for a year or less. Fewer than 5% planned very short stays abroad and to retain their positions in Poland.

Concerning the main pull factors, 78% of students indicated higher salaries, while 75% mentioned better working conditions; 66% indicated the opportunity to gain new experiences as the main pull factor, while 58% identified better professional stability (no risk of unemployment or no need for frequent changes of the employer) as the main motivation. Almost half of the respondents indicated lack of professional perspectives in Poland as a push factor.

72% of students had already undertaken concrete steps to accomplish their migration goals, including enrolling in intensive language courses (35%), browsing employment advertisements (18.6%), and establishing contacts with other migrants (15.3%). 4% already spent time abroad (e.g. completing internship) and 3.6% consulted recruiters, but only 0.8% applied for work abroad via an employment agency.

The correlation coefficient for the probability of migration and preparations to leave (the variable is a sum of all ‘yes’ answers to possible actions) was 0.28. Respondents were also asked what might affect their plans. Higher remuneration (79.2%) and improved access to specialty training (78.5%) were the main factors mentioned. The likelihood of getting a postgraduate position in Poland and family reasons followed. Interestingly, the vast majority of respondents (84.2%) thought that professional development opportunities do currently exist in Poland. Additionally, 41.7% of students stated that employment conditions in Poland were satisfactory.

### The profile of a potential emigrant

**Figure 1:**
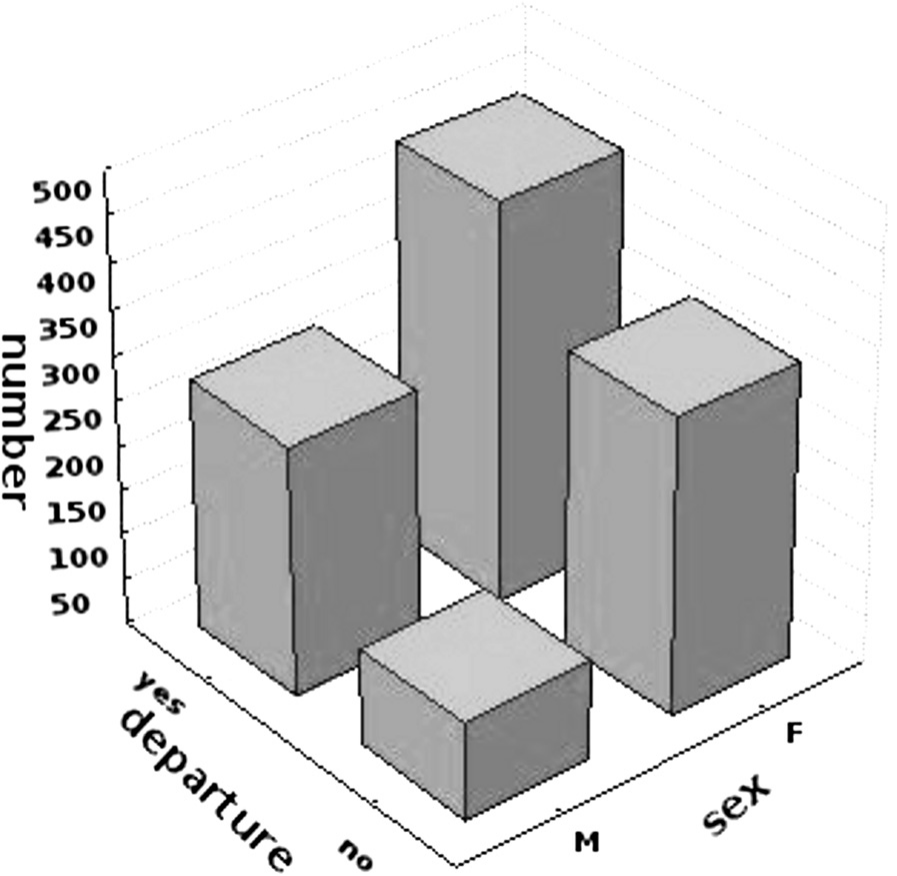
The correlation between gender and willingness to emigrate.

On average, the respondents had an average rating (GPA) during their studies of 3.93 ± 0.42. The commonly applied rating in Poland consist of 6 grades, with 2 (non acceptable) as the lowest, 3 (acceptable), 3.5 (quite good), 4 (good), 4.5 (better than good) as the middle grades, and 5 (very good) as the highest. We found no significant differences in grades between those contemplating migration and those planning on remaining in Poland. As presented of the Figure 1, there was a significant relationship (*P* = 0.0286) between gender and desire to migrate, although its strength was negligible, as evidenced by Φ2 = −0.087 and τ-b = −0.087 (see Figure 1).

There was a statistically significant association (*P* < 0.0001) between the year of study and plans to migrate but this was also very small (Φ2 = −0.1429 and τ-b = −0.1429).

Table 1 presents the parameters of a high relevance logistic regression model to explain the phenomenon of taking the decision to emigrate (*P* = 0.0000). The odds ratio of units, and the *P* level indicate the important role of age and gender in explaining the decision-making process. The results show that while being a year older, the willingness of an examined person to take the decision to leave is reduced by 16.94% if a person to decide to emigrate is a woman; the likelihood to leave is reduced by 49.52%. The model is significant in describing the phenomenon (*P* < 0.0001). However, its explanatory power is not satisfactory, since it can only classify 63.2% of cases correctly.

**Table 1:** Odds ratio estimates for emigration preferences by age and gender

**Emigration**	**Intercept**	**Age**	**Gender**
estimation	74.481 9	−0.185 6	−0.683 6
Standard error	25.307 0	0.053 1	0.2461
t(1059)	2.943 1	−3.497 7	−2.778 2
p level	0.003 3	0.000 5	0.005 6
Wald’s Chi-square	8.662 0	12.234 2	7.718 5
p level	0.003 3	0.000 5	0.005 5
Odds ratio of unit		**0.830 6**	**0.504 8**
Model significance		*P* = 0.000 0	

**Figure 2:**
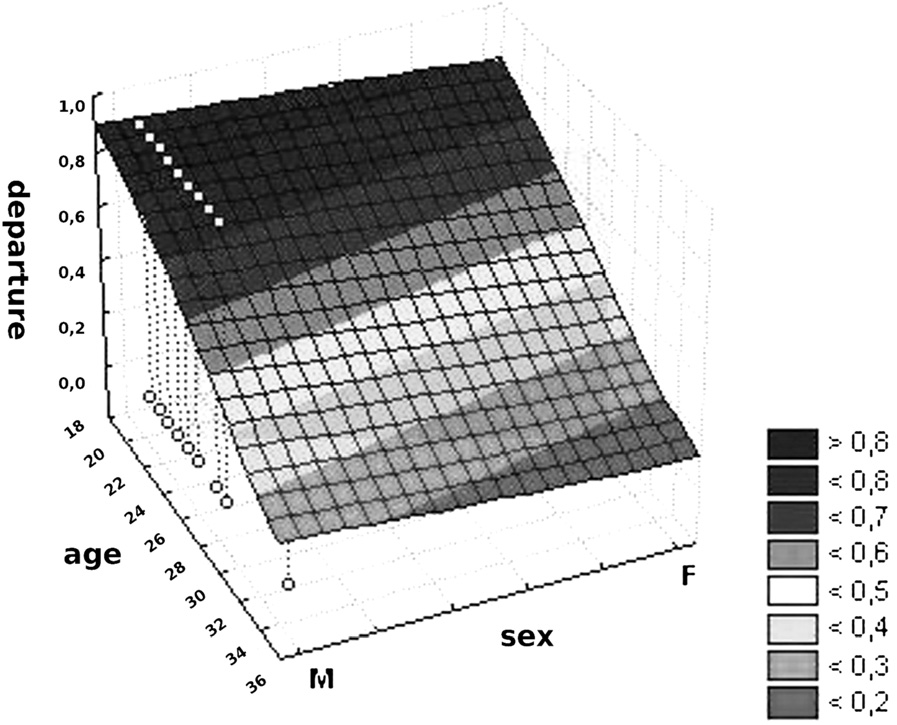
The correlation between gender and age and the willingness to emigrate.

Based on the logistic regression, we can predict that the probability to emigrate decreases with age; for men it decreased by 17%, and for women by 50% between Year 5 and Year 6 of medical school. Figure 2 presents the correlation between gender and age and the desire to emigrate.

Log-linear analysis was used to analyze a number of other important variables, including year of study (penultimate or last); perception of career opportunities in Poland (yes/no); and possibility of obtaining satisfactory working conditions in Poland (yes/no). The variances for each of the analysed features grouped with regard to the decision to move (using the F test and significance level 0.05) were also compared. Significant differences of variance in the case of features—such as satisfaction with job conditions in Poland; development opportunities in Poland; gender; and year of study—were observed.

Data analysis determined the key variables that explain the planned length of emigration. The length of emigration variable depends on the year of study (penultimate or last), place of origin (rural, urban, large city or small town), perceptions of professional development opportunities in Poland (yes/no), and ability to find satisfactory employment (yes/no). Preliminary analysis showed that migration decisions were affected by all of the aforementioned factors.

### Projections on the scale of emigration

Based on the survey results we can forecast that 26-36% of Polish medical students will emigrate in the next few years. Such an estimate results from multiplying the percentage of people declaring a desire to migrate with the likelihood of departure they declare. In addition, differences in the propensity to leave between men and women were taken into account. Of the study participants, 62% estimated the likelihood of migration at 50%. Additionally, 27.5% of the respondents named the target country. Students in their penultimate year of study declared a stronger desire to migrate than final year students. At the same time, many students were optimistic about career opportunities in Poland and satisfactory working conditions: 84.2% and 58.3%, respectively. However, a considerable number of respondents (51.9%) indicated that while obtaining satisfactory remuneration is possible in Poland, achieving it requires major efforts such as working in a number of parallel positions and/or working overtime). Of the respondents, 80% believed that healthcare reform (with no clarification of what kind of reform they expect) is essential if they are to stay in Poland.

## Discussion

Our research corroborates findings of previous smaller surveys, including a study conducted at the Medical University of Silesia in 2006 [21] and two surveys carried out by the Hippocrates Medical Association at the Jagiellonian University in Cracow in 2007 and 2008 [22]. Compared to the present survey a significantly higher percentage of students (85%) declared a desire to migrate in the Silesian study in 2006.

Not surprisingly, older students were less willing to migrate than younger ones. As indicated before, students continue to modify their migration plans throughout the course of their studies; as they near graduation they are better equipped to assess their realistic employment prospects. Additionally, the 2006 study identified similar reasons for migration, similar migration plans, target countries (indicating such countries as United Kingdom, Germany or Norway as the most desired) as well as the expected time of planned residence abroad.

In case of the Cracow study, slightly different results were observed in relation to the basic question concerning migration plans. In 2007, 65% of respondents declared plans to migrate, while in 2008 this percentage dropped to 42%. The percentage of people declaring no migration plans – 37% — is consistent with the current study. Distinctly different were results regarding motivations of students who did not wish to migrate. Family reasons dominated in both studies; however, in the present multi-site survey a significantly higher percentage of students did not identify access to satisfactory employment in Poland as their main motivation to remain in Poland. In the previous study, most frequent responses included application to postgraduate programs and patriotic considerations; these motivations were absent in the current multi-site study. Furthermore, only 2% of respondents in previous studies indicated insufficient ability to speak a foreign language as a barrier to migration, while 11% of students in the current study mentioned lack of language abilities.

A study regarding migration plans of Estonian physicians after the accession to the European Union (EU) in 2004, indicated a considerable “threat” of an outflow of medical doctors, mainly to Scandinavia. At the same time, the findings indicated that the percentage of Estonian physicians with migration plans was lower than in other countries in the region, amounting to 5.4%. Similar surveys conducted in Central and Eastern Europe found that 10.4% of Polish, 15.6% of Czech and as many as 24.7% of Hungarian physicians contemplated migration in 2004 [23]. Different estimates put the number of Estonian doctors wishing to emigrate within the first two years of EU membership at 5% [24]. The same study estimated that 3.1% of Lithuanian physicians and 7.7% of Polish anesthesiologists migrated during this time period [25]. However, the comparability of these figures with those of the present study is limited due to the fact that all cited studies were limited to dichotomous questioning of whether doctors planned to emigrate or not and did not attempt to estimate the likelihood of migration.

Research conducted in 2004 in Croatia, which is not an EU Member State, found that 71% of medical students wished to migrate if they could not attend specialization training at home. Similarly to participants in the present study, Croatian students indicated low income, problems in obtaining employment, and a poor healthcare system as push factors. Noteworthy was the fact that Croats mentioned neighboring Slovenia as the most desired destination country, which offers cultural and geographic proximity and a higher level of economic development [26].

Preferences towards emigration among students might change once they enter the labour market, as the probability of not obtaining a specialty training position in Poland is not high and would not have been a major determinant of migration preference in the present study. Selection into prestigious specialty training programs is highly competitive but overall there are enough postgraduate specialty training places. This is also in concordance with our finding that academic results in medical school do not correlate with migration preferences.

## Conclusions

The majority of Polish students considering migration see it as a serious alternative to the continuation of their professional training. If this trend continues, it can pose a serious threat to the Polish health care system. On the other hand, the observed decline of the interest in leaving among final year students compared to those in their penultimate year of study, the moderate involvement in concrete departure plans and the generally optimistic views about the opportunities for professional development in Poland suggest that the actual scale of brain drain of young Polish doctors due to emigration will be more limited than previously feared.

## Competing interest

The authors declare that they have no competing interests.

## Authors’ contributions

KK-S conceived the study, provided the design, coordinated the research process and contributed to the interpretation of the results and discussion, AS made the statistical analysis and prepared the framework of the results section, PR drafted the manuscript, provided the core of the discussion and contributed to the results section, CG helped to draft the manuscript, consulted the results and discussion, as well as provided general suggestions regarding the design of the study; AS contributed to the results section and conclusions, KK contributed to the results section and conclusions. All authors read and approved the final manuscript.

## Source of financial support

Ministry of Health of the Republic of Poland under agreement No. 11/EKS/2008/1204/3748/MNiSzW.
